# Designs for Evaluating the Community-Level Impact of Comprehensive Prevention Programs: Examples from the CDC Centers of Excellence in Youth Violence Prevention

**DOI:** 10.1007/s10935-016-0425-8

**Published:** 2016-03-09

**Authors:** Albert D. Farrell, David Henry, Catherine Bradshaw, Thomas Reischl

**Affiliations:** Department of Psychology, Virginia Commonwealth University, P.O. Box 842018, Richmond, VA 23284-2018 USA; University of Illinois at Chicago, Chicago, IL USA; Johns Hopkins University, Baltimore, MD USA; University of Michigan, Ann Arbor, MI USA

**Keywords:** Violence prevention, Research designs, Community interventions, Interrupted time series, Multiple baseline

## Abstract

This article discusses the opportunities and challenges of developing research designs to evaluate the impact of community-level prevention efforts. To illustrate examples of evaluation designs, we describe six projects funded by the Centers for Disease Control and Prevention to evaluate multifaceted approaches to reduce youth violence in high-risk communities. Each of these projects was designed to evaluate the community-level impact of multiple intervention strategies to address individual and contextual factors that place youth at risk for violent behavior. Communities differed across projects in their setting, size, and how their boundaries were defined. Each project is using multiple approaches to compare outcomes in one or more intervention communities to those in comparison communities. Five of the projects are using comparative interrupted time-series designs to compare outcomes in an intervention community to matched comparison communities. A sixth project is using a multiple baseline design in which the order and timing of intervention activities is randomized across three communities. All six projects are also using regression point displacement designs to compare outcomes within intervention communities to those within broader sets of similar communities. Projects are using a variety of approaches to assess outcomes including archival records, surveys, and direct observations. We discuss the strengths and weaknesses of the designs of these projects and illustrate the challenges of designing high-quality evaluations of comprehensive prevention approaches implemented at the community level.

## Introduction

A growing appreciation of the multiple influences that promote and sustain youth violence has led to repeated calls to move beyond interventions that focus on narrowly-defined sets of individual-level risk factors by developing more comprehensive approaches that address multiple domains of risk (Farrell & Camou, 2006; Gottfredson, 2001; United States Department of Health and Human Services [USDHHS], 2001). The Centers for Disease Control and Prevention (CDC) promoted this broader focus in 2010 by funding six National Centers of Excellence in Youth Violence Prevention (YVPCs) to evaluate multifaceted approaches to youth violence prevention (Matjasko, Massetti, & Bacon, 2016). Researchers at each center proposed intervention strategies and evaluation designs that met several specific criteria. Intervention strategies had to: target high-risk communities, include components targeted at both the general population of the community and individuals at elevated risk for violence, be evidence-based, and focus on risk factors representing multiple levels of influence (e.g., individual, relationships with parents or peers, and the community) (see Kingston, Smokowski, Sutherland, & Sullivan, 2016 for details regarding the interventions). Evaluations had to be designed to provide a rigorous test of the impact of the interventions on changes at the community level. The six YVPCs are being conducted by researchers at Johns Hopkins University (JHU), the University of Chicago (UC)/University of Illinois at Chicago (UIC), University of Colorado at Boulder (CU-B), University of Michigan (UM), University of North Carolina at Chapel Hill (UNC), and Virginia Commonwealth University (VCU). This article describes key opportunities and challenges to developing research designs for evaluating these comprehensive approaches and the methods used by the YVPCs to address these challenges.

The CDC specified several requirements for the designs to be used in these evaluations, with the overall goal of providing a rigorous evaluation (CDC, 2010). A key requirement was that projects implement the comprehensive intervention strategy in a high-risk community with one or more appropriately matched comparison communities. Projects were also required to have a plan for detecting community-level changes in youth violence outcomes over time. Although the primary focus was on the overall impact of the comprehensive approach, the CDC also encouraged projects to evaluate the impact of specific intervention components. Projects also had to be feasible within a 5-year period and available budgets. Although there are numerous examples of the use of research designs, such as randomized controlled trials, for evaluating the impact of more narrowly focused interventions such as school-based interventions (e.g., Wilson & Lipsey, 2007), there are far fewer examples of designs to evaluate the community-level impact of more comprehensive approaches (see Biglan, Ary, & Wagenaar, 2000).

The designs used by the six YVPCs, and the manner in which they attempted to meet the challenge of rigorously evaluating the community impact of prevention efforts, illustrate issues relevant not only for those in the field of youth violence prevention, but for others attempting to evaluate comprehensive interventions implemented at the community level. This article discusses the approaches taken by the six YVPCs to address the following related sets of issues: (a) defining and selecting communities for inclusion, (b) developing an appropriate research design, and (c) identifying measures that assess community-level outcomes. We conclude with a discussion of the strengths and weaknesses of the designs that were used, and areas in need of further development.

## Defining and Selecting Communities

The CDC specified that projects focus on high-risk communities, with community defined as “individuals residing in a geographical area such as a catchment area or a neighborhood” (CDC, 2010, p. 6). This focus was based on the assumption that key causes of violent behavior include systemic factors in settings where youth live. This broad definition raised some challenging questions. What is a community? How are its boundaries defined? What is a meaningful unit for studying change? Defining the unit of analysis for each project was largely determined by the focus of prevention activities, which, in turn, was influenced by the theory of change underlying the intervention. The setting, size and defined boundaries of the communities targeted by the YVPCs for their intervention and evaluation efforts differed considerably. Five of the projects focused on urban communities. These included a mid-sized urban area (Flint, Michigan), a larger urban area (Richmond, Virginia), and three major metropolitan areas (Baltimore, Maryland; Denver, Colorado; Chicago, Illinois). In contrast, the YVPC at UNC focused on a large rural county in North Carolina (see Table 1).

**Table 1:** Overview of projects evaluating comprehensive prevention efforts to reduce youth violence

Center	Research setting	Units of analysis	Unit selection criteria	Comparison site selection	Design(s)
University of Chicago (UC)/University of Illinois at Chicago (UIC)	Chicago, IL	Neighborhoods—police beats	Crime rates Demographics Community resources	Matched neighborhoods	Interrupted time series with matched comparison communities; regression point displacement design
University of Colorado-Boulder (CU-B)	Denver, CO	Neighborhoods	Crime rates Demographics Community resources Planned activities	Random assignment of matched neighborhoods	Main analysis: Two group pre–post design. Secondary analyses: Interrupted time series with randomly assigned communities
Johns Hopkins University (JHU)	Baltimore, MD	Neighborhoods	Crime rates Demographics Social characteristics Community resources	Matched neighborhood	Interrupted time series with matched comparison communities
University of Michigan (UM)	Flint, MI	Neighborhoods	Crime rates Demographics Community resources	Matched neighborhood	Interrupted time series design; two-group comparison design with one or more pretests and posttests; one-group design with a pre- and post-test
University of North Carolina at Chapel Hill (UNC)	Robeson County, NC	Counties	Crime rates Racial diversity Rural region	Matched county	Interrupted time series with matched comparison communities; regression point displacement design
Virginia Commonwealth University (VCU)	Richmond, VA	School attendance areas	Crime rates Demographics Community resources Size Planned activities	Random assignment of three school attendance zones	Multiple baseline design with randomization; regression point displacement design

The process used to define and select communities within these settings was guided by multiple criteria specified in the CDC’s funding announcement. Communities were identified that were high risk based on surveillance data and input from community partners. This ensured that the communities with which the centers partnered were characterized by a high level of need based on data, and that there were opportunities to demonstrate reductions in violence in response to prevention strategies. The definition of communities also had to be consistent with the focus of the intervention components. In order to ensure that the interventions had a reasonable likelihood of affecting the targeted outcomes, communities needed to be large enough to capture the context that contains the factors targeted by the intervention (e.g., community-level social capital), yet not so large that the available resources were insufficient to implement interventions with adequate coverage and dosage. Most of the YVPCs chose areas with infrastructure and community resources that could be mobilized and in some cases leveraged to address youth violence. From a design standpoint, communities needed to be reasonably autonomous (e.g., not share resources such as recreational programs and community centers) and sufficiently distant from their comparison communities to minimize potential diffusion of intervention effects. How communities were defined differed based on specific factors related to each project (see Table 1). For example, communities in Richmond were defined by school attendance zones because a school-based intervention was a key intervention component, and high-risk neighborhoods within zones were identified for more intensive community intervention efforts. Communities in Chicago were defined by police beats because they represented the unit by which crime data were aggregated. Researchers at UNC were particularly interested in studying youth violence prevention in a racially diverse rural setting and focused on an entire county where 39 % of residents were American Indian, 25 % were African American, and 33 % were White. The remaining three projects defined communities based on census block groups or neighborhood units defined by city planners.

The design of each project necessitated identifying intervention and comparison communities that were as similar as possible in terms of violent crime rates, community demographics, risk factors, and community resources. The research teams used a variety of approaches to select these communities. Most reviewed community surveillance data and resources to identify comparison communities that were as similar to the intervention community as possible. CU-B used cluster analysis to group similar neighborhoods in Denver into clusters based on their social characteristics and crime rates. In some cases, identifying comparable comparison communities proved challenging. This was particularly true for the UNC project, which focused on a population unique to the State that included a high percentage of American Indians. Although researchers at UNC were able to identify an adjacent rural county that was similar in terms of low socioeconomic status, it had lower rates of violence and did not have a large American Indian population. For each project, the researchers identified comparable communities based on data available at the start of the project, with the caveat that changes in demographic and other community characteristics might occur during the course of the 5-year projects that may affect the comparability of communities. All of the projects are therefore monitoring violence prevention efforts and other community dynamics that could influence violent crime during the studies.

## Research Designs

Although there were some similarities, each project pursued a slightly different approach to meet the challenge of developing a rigorous evaluation design. Several factors limited the range of potential designs. Each project implemented multiple intervention strategies designed to address both individual and contextual factors that place youth at risk for violent behavior. This necessitated focusing on communities rather than individuals as the unit of analysis. Evaluating change at this level coupled with budget constraints precluded designs such as randomized trials that are frequently viewed as the gold standard for evaluating interventions (Altman et al., 2001; Flay et al., 2005). Because of the focus on communities as the unit of analysis, this would have required a large sample of communities to provide adequate statistical power to detect intervention effects (Murray, 1998). Although there are some examples of large-scale cluster randomized trials in which communities have been randomly assigned to intervention and control conditions (e.g., Brown, Graham, Hawkins, Arthur, & Baldwin, 2009; Chamberlain et al., 2012), this strategy was not feasible given the resources available for these projects. In addition to resource considerations, cluster randomized trials may not be well suited to evaluations of community-level interventions (e.g., Biglan et al., 2000; Brown & Lilford, 2006; Hawkins, Sanson-Fisher, Shakeshaft, D’Este, & Green, 2007). Biglan et al. (2000), for example, have argued that randomized trials do not provide an adequate basis for understanding or addressing contextual factors that influence the success of intervention strategies. For example, a family intervention component being implemented by the VCU project requires the recruitment of high-risk students and their families. Initial efforts to recruit families during the first year of implementation had limited success. Discussions between the research team and school staff and administration led to the development of alternative strategies that improved recruitment rates. The design of the VCU project, which staggers implementation of the intervention across three communities, enables the investigators to incorporate these strategies into their recruitment efforts as they begin implementation in each community. It also allows them to tailor some strategies to address contextual factors that become evident during their work within each community. This approach differs dramatically from a randomized trial in which a single, standardized strategy would be simultaneously implemented in a large number of communities assigned to an intervention condition.

Most of the YVPC projects are using multiple approaches to investigate intervention effects. Each includes a design that compares changes in the intervention community to a comparison community. Most projects are also comparing changes in the intervention community relative to a broader set of communities. Several projects also are attempting to evaluate the impact of specific intervention components.

### Comparisons of Intervention and Matched Control Communities

A primary focus of each project is to contrast outcomes within the intervention community to outcomes in the comparison communities (see Table 1). Five of the projects are using a comparative interrupted time series design, and one is using a multiple baseline design. Three projects are also using pretest–posttest comparisons to analyze a subset of outcomes.

#### Comparative Interrupted Time Series Design

Five projects (JHU, UC/UIC, CU-B, UM, and UNC) are using a comparative interrupted time series design (Shadish, Cook, & Campbell, 2002) to compare outcomes in the intervention community to one or more comparison communities. This design involves repeated measurement of outcomes in the intervention and comparison communities and is typically used for quasi-experimental designs in which communities are not randomized to conditions. Program effects are evaluated by comparing changes in key outcome indicators before and after implementing the intervention in the intervention community with changes during the same period of time in matched comparison communities. Interrupted time series designs differ from pre–post designs that typically rely on only a few pretest observations in that they require multiple observations both before and after initiating intervention activities. This is critical to determining if the trend during the intervention phase deviates from the trend during the baseline phase. A primary focus of the YVPCs is on tracking changes in community levels of violence based on surveillance data from sources such as police records, school system data, hospital records, and health departments (Masho, Schoeny, Webster, & Sigel, 2016). The fact that these data are routinely collected provides the projects with multiple years of baseline data. For example, the UNC and UC/UIC projects have data going back to 2002. Moreover, because some archival data are continuously collected it is possible to aggregate data into multiple observations per year. For example, the UC/UIC project will examine changes in monthly rates of violence-related offenses based on police records. Several projects have also developed partnerships that provide access to other relevant data. For example, JHU has access to data from a school climate survey administered annually in the Baltimore City Public Schools that will provide 7 years of baseline data.

Interrupted time series designs are a useful option for analyzing changes within an individual unit when multiple points of measurement are available. Linear models with random effects provide a flexible approach that can be shaped to fit multiple longitudinal study designs and data collection schedules (Hedeker & Gibbons, 2006; Singer & Willett, 2004). These analyses employ models that assess the effects of introducing an intervention on the level (intercept) and change (slope) of the outcome variable. This basic longitudinal model may also be modified to compare changes in the levels and slopes in the intervention and comparison communities during the baseline phase, and again following introduction of the intervention. Such a design does not require that the points of measurement be regularly spaced and this type of analysis can be used with distributions representing binary or count data as well as continuous outcome measures. Random effects models can be used to accommodate multiple levels of analysis (i.e., times within individuals, individuals within communities).

#### Multiple Baseline Design

VCU is using a multiple baseline experimental design (Biglan et al., 2000) to evaluate their intervention strategy. This design has also been referred to as a stepped-wedge trial (Brown & Lilford, 2006). Multiple baseline designs involve continual collection of outcome data with multiple units, in this case communities, randomly assigned to receive the intervention at different points in time. Analyses are then conducted to determine if the introduction of the intervention within each community is associated with subsequent changes in outcome measures. This design differs from the comparative interrupted time series design in that each community receives the intervention, with the order and timing of initiating the intervention determined randomly. The focus of the VCU project is on three communities defined by school attendance zones. Based on random assignment the intervention was initiated in one of the communities in Year 2, a second community in Year 3, and the third community will begin receiving the intervention at the end of the project. As with the other projects, the VCU project is able to take advantage of multiple years (i.e., 10) of data on community indicators to assess intervention effects. The investigators are also collecting four waves of data per year based on outcome measures being completed by adolescents and teachers within the middle school in each community.

The linear model approach described for comparative time series designs can be expanded to analyze the outcomes of multiple baseline designs (Ferron, Bell, Hess, Rendina-Gobioff, & Hibbard, 2009). One difference is that multiple baseline designs provide estimates of the intervention effect (i.e., phase) for each community. Intervention effects can also be evaluated using a randomization test (Edgington & Onghena, 2007). This involves comparing the treatment effect (e.g., difference between the intervention and control phases) obtained from the design that was actually used based on the randomization of order and timing, to the distribution formed by calculating the effect for each of the possible combinations of order and timing that could have been selected based on the randomization scheme. For the VCU project this included 24 possible patterns. Randomization tests can be used with virtually any statistical procedure, are distribution-free, work well with small samples, and require few assumptions. Their disadvantages are that they do not provide interval estimates for the size of the treatment effect, a model for how this effect changes over time, or an estimate of the effect for a particular community. VCU will be using these analyses in addition to linear models.

#### Pretest–Posttest Comparison Design

Three projects are supplementing their primary analyses with analyses comparing pretest-to-posttest changes in intervention and comparison communities on outcomes for which archival data are not available. CU-B collected pretest data in intervention and comparison communities using community surveys to obtain youth and adult reports of violence, victimization, and risk factors during the year preceding implementation of the intervention, and will be comparing these to data they plan to collect 4 years later at the end of the project. UM will examine pre-to-post changes in the intervention and comparison community on surveys of neighborhood residents (two pretests and two posttests) and on observer ratings of property maintenance (one pretest and three posttest observations). Finally, UNC is collecting data on student reports of their frequency of violent behavior and perceptions of neighborhood crime at the end of each school year in 20 schools in the intervention county and 8 schools in the comparison county. This will enable them to compare trajectories on these outcomes across the 5 years of their study.

Most of the projects will use multilevel models or analyses of covariance to analyze the data from these pre-to-post designs. The investigators will then employ multilevel models when there are multiple levels of nesting (Hedeker & Gibbons, 2006; Singer & Willett, 2004). For example, UNC investigators are collecting data from individual students from 28 schools within their intervention and comparison communities. CU-B and UM will use basic analysis of covariance models to compare pretest and posttest data obtained from observations based on multiple residents or property assessments within intervention and control communities.

### Comparisons With a Broader Set of Communities

Each project is supplementing the designs described in the preceding section with analyses comparing changes in intervention communities to changes within a broader set of communities. Having multiple comparisons generally leads to more robust estimates of program effects (Rosenbaum, 2009). Although, the inclusion of a broad set of comparison communities provides better control for other factors that might impact the observed outcomes, a critical issue concerns the selection of comparison communities. In particular, designs that select comparison communities that are similar to intervention communities on key demographic and geographical variables are more likely to approximate the findings of experimental designs (Cook, Shadish, & Wong, 2008). All six projects will be using a regression point displacement design (RPDD; Linden, Trochim, & Adams, 2006), which is a variant of the regression discontinuity design that is appropriate for evaluating interventions administered to a single community. Analysis of RPDD data is based on simple linear models that may be enhanced with distributions appropriate to binary or count data, and the use of propensity scores to adjust for non-randomness in selecting the intervention community. The power of this design is strongly dependent on the correlation between pretest and posttest measures, which may vary according to the particular indicator chosen (e.g., homicide vs. overall violent crime) and the level of aggregation (i.e., census tract, block, police beat, community). The intervention effect in an RPDD analysis is the difference between the actual posttest score of an intervention unit, and the predicted posttest score based on the pretest and posttest values of other similar units. The projects are using these designs to conduct analyses of surveillance data collected within the communities where these projects are being conducted.

The UC/UIC investigators are using an RPDD approach to compare changes in incidents of violent crime based on police reports within the police beats in their target community to all the police beats in Chicago. They will also compare rates to police beats in Chicago communities implementing CeaseFire (Skogan, Hartnett, Bump, & Dubois, 2008), which represents one of their primary intervention components. This approach will strengthen the design by allowing comparisons to a set of similar communities. The UNC investigators will compare changes in the county where they are implementing interventions to all 99 other counties in the state of North Carolina. They will also compare changes in school disciplinary indicators and achievement for 21 schools in their intervention community to a subset of 60 matched comparison schools. The VCU investigators will use an RPDD approach to compare trends in census block groups within the intervention community to other census block groups within the City of Richmond that show similar baseline rates of violence-related incidents based on surveillance data.

The JHU investigators are using RPDD to compare changes in police and school data in the intervention community to changes in a larger set of comparison communities that are most similar to the intervention community on multiple measures of violence and other characteristics (e.g., social distress). They will also compare changes in their intervention community to those in a smaller set of comparison neighborhoods identified a priori, based on similar community demographic characteristics and crime rates. In Baltimore, there has also been a roll out of the community-level CeaseFire/Cure Violence model in other communities led by the health department. This allows for a contrast between the CDC-funded Baltimore CeaseFire site (called *Safe Streets* in Baltimore; Webster, Whitehill, Vernick, & Curriero, 2013), the health department sponsored sites, and other matched controls. The UM YVPC will use police incident data (dating back to 2005) to compare violent crime trends in the intervention region and a similar comparison region both before and after the start date of the multiple interventions. The CU-B investigators will use a similar analysis to compare the intervention community with a set of all similar communities included in the same high risk cluster of communities identified in their original selection of an intervention and comparison community using archival police arrest data.

In addition to using an RPDD approach, the UM researchers will use spatial analysis to compare changes in the intervention community to changes in all 39 populated census tracts within the City of Flint, controlling for environmental and socio-demographic variables (e.g., population size, poverty rate, racial composition). This will enable them to examine diffusion of intervention effects by determining the extent to which census tracts adjacent to the intervention community experience some benefit that decreases as a function of their distance from the intervention community. The spatial analysis approach allows the researchers to model the radiating effects of the interventions while controlling for the spatial autocorrelation (dependency) problem often encountered in spatial regression models. Spatial regression analyses that fail to compensate for autocorrelation can produce unstable parameter estimates and unreliable significance tests (Haining, 2003).

### Examining the Effects of Individual Intervention Components

Although the primary focus of these projects is on evaluating the overall effects of the comprehensive interventions implemented by each project, there is also interest in determining the impact of the individual components that constitute these interventions. This provides a test of each intervention’s logic model and information that could ultimately improve the efficiency of the overall intervention approach by eliminating or enhancing individual components that do not produce desired results. Although each component in the overall intervention was required to be evidence based, it is still useful to determine the degree to which they contribute to any overall effects. Ideally, projects would evaluate each intervention component using dismantling-treatment or constructive-treatment strategies in which the contribution of individual components to an overall intervention package are determined by comparing groups that received different sets of components (Kazdin, 2003). Such designs, however, are more feasible when individuals rather than communities are the focus of interventions. Nonetheless, four of the projects are attempting to examine the impact of specific intervention components.

Annual surveys of students in schools within the intervention and comparison counties conducted by UNC include measures of social environmental risk and promotive factors targeted by their intervention. These outcomes will be analyzed across the 5 years of the project using hierarchical linear modeling to determine if students from schools in the county where the school intervention is implemented show the expected pattern of change relative to schools in the comparison county. The collection of data from a large number of schools (i.e., 28) allows modeling of the effects of the intervention condition at the school level, and dosage (i.e., number of years of exposure to the intervention) at the student level, while controlling for student and school-level covariates.

Researchers at VCU are collecting data on measures of school climate every 3 months during each school year from a random sample of students and teachers in middle schools in their three participating communities. They are also collecting four waves of data each year from a random sample of students in each school on constructs targeted by the school intervention (e.g., achievement motivation, peer norms, self-efficacy for nonviolence). They will examine trends in these outcomes across the 5 years of the project to determine if the school intervention produces the expected pattern of change as the intervention is initiated in each school. The VCU researchers are also evaluating the impact of a family intervention component on families of high-risk youth by collecting pretest and several waves of posttest data from adolescents and their parents on variables specifically targeted by the intervention. Measures include adolescent reports of problem behaviors and family variables (e.g., parent–child communication and relationships), parental ratings of child behavior and family and parenting variables (e.g., parenting practices, parent–adolescent communication), and an observational measure of parent–adolescent interactions. These data are collected from families of adolescents meeting criteria for referral to the intervention in schools where the intervention is being implemented during a given year to their counterparts in schools where the intervention has not yet been implemented. Similarly, JHU is using a pre–post design to examine the added benefits of exposure to Coping Power (Lochman & Wells, 1996), an indicated preventive intervention being implemented in the schools to determine the added benefit of involvement in this program relative to the other school-wide approaches used with all students (e.g., Positive Behavioral Interventions and Supports; Sugai & Horner, 2002).

The UM investigators are also conducting analyses of the effects of all six programs in their broad intervention strategy. For example, they will evaluate the effects of two of the interventions, *Project Sync* (a brief motivational interviewing intervention for youth presenting in a hospital emergency department) and the Fathers and Sons program (a 10-session positive health behavior intervention for African American boys and their fathers; Caldwell et al., 2004) by comparing outcomes for youth in the intervention community to those in a control group of youth in the comparison community. They will also examine the effects of their place-based intervention, the LandBank’s Clean and Green program, by comparing direct observations of maintenance on foreclosed properties in the Clean and Green program to observations of properties not involved in the program. The effects of the Community Engagement intervention on the residents’ perceptions of their neighborhoods and relationships with police will be assessed by comparing survey responses of community residents in the intervention and comparison regions.

The UC/UIC investigators are conducting analyses of archival data for police beats across the city of Chicago to determine the extent to which their overall intervention approach, which includes the CeaseFire intervention (Skogan et al., 2008) and several additional components, improves upon CeaseFire without the additional components. More specifically, they will compare data on police beats within their intervention community to other police beats that are implementing CeaseFire during the same period of time, and to other police beats that are not.

## Assessment of Outcomes

Investigators associated with each project are collecting data on a variety of outcomes to assess the impact of their intervention activities. Data sources fall roughly into three types: archival records, surveys, and direct observations. They selected outcome measures based on the goals of the evaluation, the research design, and key characteristics of the interventions being implemented. This section briefly discusses some of the factors they considered in selecting outcome measures and provides an overview of measures being used by each project. Masho et al. (2016) provide a more detailed description of outcomes and associated issues elsewhere in this special issue.

### Archival Data

A key requirement for all projects was that they evaluate the community-wide impact of their intervention efforts on youth violence outcomes over time. This was due to the emphasis of the YVPC Program on demonstrating the impact of prevention approaches on community rates of violence, thereby demonstrating the reach of the interventions evaluated. All six projects are addressing this outcome by examining changes in police data on violent crimes (e.g., homicides, shootings, assaults) committed by juveniles. Several projects are supplementing these outcomes with archival data from other sources (see Masho et al., 2016). JHU will examine 14 years of data from the school system on disciplinary referrals and suspensions for fighting and weapons incidents, and 11 years of data from an annual survey of students’ perceptions of school climate. The UM project will have access to 10 years of data on violence-related injuries based on hospital emergency department data. The VCU project will examine up to 15 years of data on violence-related injuries based on both hospital emergency department visits and ambulance calls, arrests for violence-related incidents based on police data, and school disciplinary incidents based on data from the Virginia Department of Education.

Archival data have several features that make them well suited to the designs being used by these projects. Because they are routinely collected, it is possible to examine baseline trends for up to 10 years preceding the starting dates of these projects. These incident data are also typically coded by date, location and age of those involved, which makes it possible to aggregate them into geographical units, time intervals, and age groups that meet the requirements of the designs being used by these projects. For example, the calculation of monthly rates of violent crime incidents involving youth within specific geographical areas (e.g., police beats, census block groups) meets the needs of comparative interrupted time series and multiple baseline designs. Because these designs focus on examining changes in trends over time, their power to detect effects rests partly on the number of assessment occasions prior to and following initiation of the interventions. Similarly, RPDD requires the collection of data from enough comparison communities to create estimates of expected posttest values (Linden et al., 2006). Moreover, the data must have been collected sufficiently prior to implementing interventions to form credible pretest measures. Finally, although pretest and posttest measures need not be identical, the power of RPDD depends on the correlation between pretests and posttests (Wyman, Henry, Knobloch, & Brown, 2015). Incident-level crime data aggregated at the community level tend to be very stable, providing an excellent basis for RPDD analyses. These data can also be stratified to examine the impact of the intervention on individuals within the age group targeted by the intervention versus changes that may reflect more general trends within a community. Finally, these data can be geocoded, which provides a basis for doing spatial analyses such as those being conducted by the UM project.

### Other Sources of Outcome Data

Most projects are supplementing archival data by actively collecting data within their intervention and comparison communities. These include measures related to primary outcomes such as violent behavior and victimization, and measures of specific constructs targeted by components of the intervention. The CU-B researchers are conducting a community survey of youth and adults in the intervention and comparison neighborhoods prior to implementation and during the final year of the project. They will also conduct school climate surveys at baseline and during years four and five. Measures include risk and protective factors at the individual, peer, family, school and neighborhood levels, and outcome measures for violent behavior, other delinquency, and violent and non-violent victimization. JHU researchers also are conducting household and street-intercept surveys three times per year to assess victimization and attitudes related to violence, and collecting observational data on residents’ exposure to violence, alcohol and other drug activity, and environmental indicators of neighborhood disorder (e.g., graffiti, vandalism), from 50 block groups in their intervention and control communities. Similar observational data are also being collected in the schools.

UNC investigators are conducting an annual survey of students at schools within their intervention and comparison counties to assess violence and other problem behavior, neighborhood crime, school climate and safety, risk factors (e.g., peer pressure, delinquent friends) and promotive factors (e.g., friend and parent support). UM is conducting neighborhood surveys of adult residents to assess neighborhood characteristics targeted by the intervention (e.g., neighborhood satisfaction, social capital, fear of crime, activism and relationship with police) every 2 years prior to and after initiating the interventions, and using observers to rate the maintenance of buildings and landscaping on property parcels (one pretest and three posttest assessments).

VCU investigators are collecting data from a random sample of students at the middle schools in their three targeted communities to assess their frequency of violent behavior, victimization, risk factors targeted by the school intervention (e.g., beliefs about fighting, peer support for aggression), and school climate; obtaining teacher ratings of aggression for these students; and collecting ratings of school climate from a random sample of teachers at each school. During each year of the project, they are collecting data from students every 3 months throughout the year and from teachers every 3 months during the school year. This will provide 19 waves of data at the school level, which is consistent with the multiple baseline design and analyses planned for these data. It also makes it possible to estimate changes across seasons of the year. This design involves planned missing data (Graham, Taylor, & Cumsille, 2001). Although four waves are collected each year, each student is randomly assigned to complete two of the waves. This strategy reduces costs, and attempts to reduce testing effects and attrition related to frequent participation. Because the resulting pattern of missing data meets the requirement of being missing completely at random, it enables the use of models such as full information maximum likelihood estimates that are able to make full use of all available observations (Shafer & Graham, 2002).

## Discussion

### Challenges

#### Internal Validity

A major concern for any project evaluating an intervention is internal validity, or the extent to which any observed changes can be attributed to an intervention rather than to other external factors (Shadish et al., 2002). A particular challenge for these six projects is the potential for selection bias. Each project is examining changes in an intervention community relative to one or more comparison communities that are either not receiving the intervention, or in the case of the VCU project, are assigned to receive the intervention on a delayed schedule. As previously noted, each project attempted to identify a comparison community that closely matched its intervention community. CU-B and VCU identified communities and randomly assigned them to conditions. The other four projects first selected the intervention community and then matched it with a comparison community. Finding an appropriate match was particularly difficult for UNC given its focus on a community that was selected not only because it had one of the highest rates of youth violence in the state, but because of its unique racial composition that included one-third American Indian, Caucasian, and African American residents. Although the investigators were able to match on several key variables such as rural setting and socioeconomic status, the comparison community’s racial and ethnic composition is quite different from that of the intervention community. Comparing changes across communities has clear advantages over examining changes within a single intervention community. However, there are likely to be some differences across communities assigned to different conditions that will complicate comparisons. Propensity score analysis can help address such initial differences. Propensity scores reflecting the likelihood of receiving treatment based on baseline variables can be incorporated in the analyses as strata, weights, or covariates, so that the analysis is more likely to reflect the net difference between treatment and comparison groups (Guo & Fraser, 2014).

The focus on a single intervention and comparison community raises the possibility that any differences that emerge during the study (or lack of differences) may be due to factors unrelated to the intervention. Comparing changes within the intervention communities to changes during the same period of time within the comparison community provides some control for broad factors that affect both communities (e.g., changes in economic conditions, city-wide policing policies). The selection of communities that display similar levels on outcomes such as rates of youth violence also provides some control for potential effects associated with regression to the mean. It does not, however, control for more isolated factors that impact only the intervention or comparison community. These may include events within either community that have a positive or negative impact on outcomes. For example, an increase in gang activity can lead to the appearance of stronger or weaker intervention effects depending on whether it occurs in the intervention or comparison community. One of the strengths of these designs is the investigators’ use of surveillance data collected over fairly long intervals of time, which will reduce the impact of any transient changes on the findings.

All of the projects are employing either an RPDD or spatial analysis to compare trends in the intervention community to a larger set of comparison communities. This strengthens the design by making it less likely that the findings are influenced by initial differences or events within a single comparison community. It also makes it possible to control for factors that vary across communities. Although this design is less susceptible to factors affecting a single comparison community, it does little to address factors that occur within the intervention community that might impact outcomes, but to the extent that historical events have an area-wide impact, effects of history should be present in the comparison as well as the intervention communities (Shadish et al., 2002). Effects of selection are more difficult for the RPDD to address, unless the selection of the targeted community is truly random.

One of the advantages of the multiple baseline design used by VCU is that it provides an opportunity to determine if the intervention effect can be replicated. Biglan et al. (2000) and others (e.g., Hawkins et al., 2007) have advocated the use of multiple baseline designs for evaluating community-level interventions. The multiple baseline design is an example of “roll-out” designs such as the dynamic waitlist (Brown, Wyman, Guo, & Pena, 2006) and stepped wedge (Brown & Lilford, 2006) designs. These designs offer better control over extraneous factors by testing intervention effects across each community at different points in time. Attributing changes in the outcome measure to an event within the community unrelated to the intervention is less plausible when a consistent pattern of intervention effects is found across replications because it is unlikely that such an event would coincide with the introduction of the intervention within each community. The community partners in Richmond have been supportive of this approach, particularly the fact that it results in each community ultimately receiving the intervention (although it occurs at the end of the funding period for one of the three communities). Furthermore, the randomization of multiple elements of the design (i.e., both the order in which the communities receive the intervention and the timing) strengthens this experimental design considerably because it increases the number of possible assignments while maintaining the systematic staggering of the introduction of the intervention (Kratochwill & Levin, 2010). Unfortunately, because of its use of a school-based intervention the VCU project had a limited number of potential patterns for randomizing the start of the intervention in the second and third communities. Because the randomly selected pattern delayed initiating the intervention in the third community until the end of the project there will not be an opportunity to see if any observed intervention effects in the first two communities are replicated in the third community during the 5-year funding period.

#### External Validity

A further challenge for each project will be determining the extent to which the findings of these evaluations can be generalized to other communities, interventions, and outcomes. Each project is implementing interventions in one or two communities. There may be unique aspects of the communities targeted by each project that influence the impact of the intervention. As we have noted, intervention communities were not selected at random. Most had high levels of youth violence, and active community organizations and community resources that could support the development, implementation, and sustainability of intervention efforts (Kingston et al., 2016). It will thus be unclear whether any observed findings can be generalized to communities with lower levels of community support or resources. Although the findings of any one project may have somewhat limited generalizability, taken as a group, their findings will represent intervention effects across a fairly diverse set of communities.

The findings of these projects will also reflect the effects of specific approaches to intervention that, in most cases, were carefully tailored to the needs of specific communities (see Kingston et al., 2016; Morrel-Samuels, Bacallao, Brown, Bower, & Zimmerman, 2016). The degree to which this occurred varied across projects, but was particularly emphasized in the CU-B project, which used the Communities That Care framework (Hawkins, 1999). This approach involved conducting household surveys to identify community risk factors and allowing community residents to select interventions from a menu of evidence-based programs. A clear strength of this model is that it may increase buy-in for community members and optimize the contextual fit of the intervention. It does, however, impact the generalizability of the findings as specific intervention activities will likely differ across communities and an intervention tailored for one particular community may be less relevant to others. This suggests that the intervention being tested may be better represented by the process that was followed in working with a community to select and implement a package of intervention strategies than in the specific interventions that were chosen (cf., Allen, Mohatt, Fok, Henry, & People Awakening Team, 2009; Beauvais, 1992; Wallerstein & Duran, 2006). More generally, projects were charged with evaluating the impact of a comprehensive prevention program that included multiple components. Although several projects, as previously discussed, are attempting to evaluate the impact of the individual interventions, it will not be possible to isolate the impact of any single component. In short, any findings will represent the overall effects of the package of interventions.

The fact that interventions are being implemented as part of a research project may also limit the extent to which the findings can be generalized. Although there is some variability across projects, most of these projects fall somewhere in between an effectiveness trial in which interventions are tested under typical real world settings, and an efficacy trial in which interventions are implemented under carefully controlled conditions (Kazdin, 2003). For example, one of the interventions being implemented in the VCU project is the Olweus Bullying Prevention Program (Olweus et al., 2007). Although a Bully Prevention Coordinating Committee has responsibility for implementing the program, the researchers are providing the committee with feedback on implementation fidelity based on classroom observations that is being used to sustain fidelity (Goncy, Sutherland, Farrell, Sullivan, & Doyle, 2015). It is unlikely that most school systems would have this level of support. Similarly, projects implementing family interventions including UNC and VCU are making intensive efforts to recruit and engage families in these interventions. More generally, projects have had to strike a balance between ensuring interventions were implemented with adequate fidelity and dosage versus determining the impact of interventions as they are likely to be implemented outside of a research context. As noted above, other communities within both Chicago and Baltimore are implementing the community-level CeaseFire/Cure Violence model, which allows for a contrast between efforts that are the focus of the YVPCs and those that are implemented independent of the project.

A significant strength of these projects is their focus on evaluating the community-level impact of the interventions through collection of multiple waves of surveillance data. Most projects are also collecting data from other sources including resident surveys, adolescent self-reports, and teacher ratings, but are not relying solely on those data. Although surveillance data also have limitations, they are not subject to many of the factors that limit the external validity of measures such as rating scales that may be highly reactive (Kazdin, 2003). The continuous collection of data also provides a more accurate assessment of changes over time. Finally, indicators such as arrest rates for violent offenses and ambulance pick-ups for violence-related injuries have considerable social validity (Wolf, 1978) and are the outcomes of primary interest to both community residents and policy makers.

#### Other Challenges

Although the collection of multiple measures of outcomes strengthens these projects, it also presents some challenges. One of the common outcome measures across the projects is police data on arrests for violent crimes committed by youth within the boundaries of the defined communities. Significantly reducing these rates is an ambitious goal as they are influenced by multiple factors, not all of which are targeted by the interventions. Moreover, not all violent crimes committed in a community are committed by residents of that community. Arrest data also represent only a subset of violent crimes that occur within any given community. An additional issue is that the time-series analyses being conducted by these projects involves aggregating these indicators of serious violent offenses into relatively small intervals of time (e.g., monthly) for defined geographical areas (e.g., census block groups, police beats, school attendance zones). At this level, the data may be overly sensitive to outliers (e.g., multiple homicides or violent crimes resulting from a single conflict, such as a territorial dispute). Some balance needs to be struck between aggregating data over time intervals small enough to generate sufficient data points for conducting analyses of interrupted time series, but large enough to ensure that they are not mostly clustered near zero. In the case of the RPDD, the correlation between pretest and posttest must be taken into account when choosing a level to which to aggregate data, because the power of the RPDD increases dramatically as the pre–post correlation exceeds .90 (Wyman et al., 2015).

Some projects are supplementing data on serious violent crimes with indicators of less serious offenses such as student reports of physical aggression and other indicators such as residents’ perceptions of neighborhood safety or teacher ratings of school climate. Although indicators of this nature are related to neighborhood violence, they may be slow to change even when actual change in crime rates have occurred. Each project is taking multiple steps to address these concerns. These include the collection of data from multiple indicators over a sufficiently long period of time to establish adequate stable estimates of pretest and posttest levels. As previously noted, most projects are also including measures of indicators specifically targeted by the intervention (e.g., neighborhood social capital, school climate, peer norms). A further limitation of these projects concerns the 5-year time frame. Although there may be some immediate impacts of an intervention on those who directly participate, community-level change may require more sustained involvement. Intervention effects are not likely to occur immediately and it is not clear precisely when such effects might emerge (Weissberg & Bell, 1997).

An additional challenge for these projects will involve the interpretation of any observed differences between the intervention and comparison communities. There are relatively few high-risk communities that are not the focus of some intervention efforts. For example, the ubiquitous prevalence of prevention programs in schools was documented by Gottfredson and Gottfredson (2001) who reported that schools in their national survey of principals implemented a median of 14 different prevention programs. However, such programs are often either not evidence-based (e.g., Ringwalt et al., 2002), or are poorly implemented (e.g., Durlak & DuPre, 2008; Halfors & Godette, 2002). Although a variety of other intervention activities are being implemented in the comparison communities, communities rarely have the resources and technical support needed to ensure high fidelity and quality of implementation (Chinman et al., 2005; Mihalic & Irwin, 2003). Comparison communities thus represent treatment as usual rather than the absence of any interventions. In some instances, participation in the project may encourage comparison communities to initiate new activities. For example, UC/UIC provided the comparison community with some findings of their community survey on risk factors. Although they did not provide assistance in interpreting the findings or initiating intervention activities, providing this information may lead to the initiation of intervention activities that would not otherwise have occurred (Shadish et al., 2002).

Most projects are making efforts to monitor intervention activities occurring in the comparison communities. For example, JHU researchers are collecting data on the quality of implementation of school-based programs in the control schools to enable them to monitor and control for potential concerns regarding contamination. JHU will also have access to data through a partnership with a community organization that will enable the investigators to track services received by youth in the intervention and comparison communities such as after-school programs, summer programs, and recreation and sports programs. UC/UIC researchers are taking advantage of data on specific communities in Chicago that are implementing CeaseFire/Cure Violence, one of the components of their intervention. Using such data allows them to compare the effects of their comprehensive intervention (including CeaseFire/Cure Violence in the high school), to other communities in Chicago that are implementing CeaseFire/Cure Violence as well as to communities that are not.

## Conclusions

Over the past 25 years substantial progress has been made in understanding what it will take to address youth violence (USDHHS, 2001). There is an increasing understanding that stand-alone programs that focus on a single aspect of this problem are not likely to be effective (Farrell & Camou, 2006; Gottfredson, 2001; Matjasko et al., 2016). In particular, there is recognition of the need to develop comprehensive programs that reduce risk factors and enhance factors that promote positive development and buffer the effects of risk factors at multiple ecological levels (Farrell & Vulin-Reynolds, 2007). The development of appropriate research designs to evaluate the comprehensive interventions that are emerging from these efforts is challenging. Early efforts to evaluate universal school-based interventions were straightforward applications of randomized trials in which classrooms were randomized to conditions in which they either received the curriculum or did not (Henry, Farrell, & The Multisite Violence Prevention Project, 2004). Increasing recognition of the influence of peers, parents, school climate, community factors, and other contextual factors influencing aggression has made it evident that implementing interventions with only a subset of students within a school is likely to achieve very limited success.

The development of school-level interventions drove the need for outcome studies in which entire schools rather than classrooms within schools represented the unit of analysis (Henry et al., 2004). The application of randomized trial designs at the school level significantly stretches the limits of resources required to implement such projects. For example, the CDC-funded Multisite Violence Prevention Project (MVPP) required four teams of researchers and the randomization of 37 schools across four sites to intervention and control conditions (Henry et al., 2004). Although the development of school-level interventions that address both individual and contextual risk factors is an important development in the field of youth violence prevention, it is clear that more comprehensive approaches are needed. Although students spend a significant portion of their time in school, they are exposed to important influences outside of school. This suggests the need for more comprehensive strategies aimed to reduce risk and enhance protective influences in multiple contexts. Designing these comprehensive efforts is challenging (Kingston et al., 2016; Matjasko et al., 2016; Morrel-Samuels et al., 2016), as is developing research designs to evaluate these efforts.

Randomized controlled trials, which have long been the gold standard for evaluating interventions, may not provide the best tool for evaluating community-level interventions (Biglan et al., 2000; Sanson-Fisher, D’Este, Carey, Noble, & Paul, 2014). Applying these designs to interventions that focus on communities requires the random assignment of a large number of communities to intervention and control conditions to achieve adequate power to detect intervention effects. This requires considerable resources that are well beyond the capacity of many funders. Implementing projects on this scale is also likely to stretch resources, which may result in compromises in critical areas. For example, limited resources for intervention activities may limit their dosage, fidelity, scope or duration. There is also a Catch-22 dilemma involved in building a comprehensive intervention: (a) Addressing youth violence requires comprehensive intervention efforts to have an impact; (b) Comprehensive intervention efforts should be constructed from individual components that have demonstrated their effectiveness; and (c) Individual intervention components are not likely to be effective unless they are part of a comprehensive intervention approach.

There is thus a clear need for initial efforts to evaluate packages of interventions before going to scale with large resource-intensive randomized trials at the community level (Biglan et al., 2000). Although smaller scale studies of individual intervention components can provide useful information about the extent to which they produce their desired effects on the specific risk factors they target, their full potential is unlikely to be achieved until they are incorporated into a more comprehensive intervention strategy. More generally, it is difficult to estimate the overall impact of a comprehensive strategy from evaluations of its components. At a more basic level, it has been argued that randomized controlled trials, which were initially developed for medical research, may be less useful for evaluating community interventions (Sanson-Fisher et al., 2014). As Biglan et al. noted, “a randomized trial is a good vehicle for testing the replicability of such principles [those that guide interventions], but it is a poor one for arriving at them” (2000, p. 33).

Youth violence remains one of the most vexing public health challenges of our time. Community desperation in the face of escalating youth violence presents a fertile field for the adoption of approaches that promise to reduce violence but may, in fact, be ineffective (USDHHS, 2001) or exacerbate the problem (Dishion, McCord, & Poulin, 1999). Aggressive marketing of untested or ineffective programs further underscores the importance of evaluating community-level approaches to reducing violence and adding to the inventory of programs with evidence for effectiveness. Although there are examples of interventions that have shown positive effects in relatively small trials, the questions of the effectiveness of violence prevention at the community level, and the likely effects, when taken to scale, of interventions that have shown efficacy in smaller studies, remain open. This article described six examples of efforts to meet the challenge of evaluating the community-level impact of comprehensive efforts to reduce youth violence in high risk communities. Each project illustrates the application of alternative designs that have been suggested for evaluating community level applications of evidence-based interventions (e.g., Biglan et al., 2000; Linden et al., 2006; Sanson-Fisher et al., 2014; Shadish et al., 2002). We hope that these examples will help inform others attempting to meet the challenge of conducting rigorous evaluations of similar approaches designed to produce community-level changes.
